# High-Performance Concrete from Rubber and Shell Waste Materials: Experimental and Computational Analysis

**DOI:** 10.3390/ma17225516

**Published:** 2024-11-12

**Authors:** Alejandra Miranda, Ricardo Muñoz, Cristopher Aedo, Flavia Bustos, Víctor Tuninetti, Marian Valenzuela, Carlos Medina, Angelo Oñate

**Affiliations:** 1 College of Engineering, Architecture, and Design, Universidad San Sebastián, Campus Las Tres Pascualas, Lientur 1457, Concepción 4060000, Chile; alejandra.miranda.ve@gmail.com; 2 Department of Mechanical Engineering, Faculty of Engineering, Universidad del Bío-Bío, Av. Collao 1202, Concepción 4081112, Chile; ricardo.munoz1701@alumnos.ubiobio.cl (R.M.); cristopher.aedo1701@alumnos.ubiobio.cl (C.A.); 3 Engineering Systems Doctoral Program, Faculty of Engineering, University of Talca, Curicó 3340000, Chile; flavia.bustos@utalca.cl; 4 Department of Mechanical Engineering, Universidad de la Frontera, Francisco Salazar 01145, Temuco 4811230, Chile; 5 Doctoral Program in Sciences of Natural Resources, Universidad de la Frontera, Francisco Salazar 01145, Temuco 4811230, Chile; m.valenzuela16@ufromail.cl; 6 Department of Mechanical Engineering (DIM), Faculty of Engineering, University of Concepción, Edmundo Larenas 219, Concepción 4070409, Chile; cmedinam@udec.cl; 7 Department of Materials Engineering (DIMAT), Faculty of Engineering, Universidad de Concepción, Edmundo Larenas 315, Concepción 4070138, Chile

**Keywords:** eco-concrete, structural concrete, porosity, decarbonization, waste valorization

## Abstract

Waste and its environmental impact have driven the search for sustainable solutions across various industries, including construction. This study explores the incorporation of solid waste in the production of eco-friendly structural concrete, aiming to reduce pollution and promote ecological and sustainable construction practices. In this context, two types of eco-friendly concrete were produced using marine shells and recycled rubber as waste materials and compared with conventional concrete through experimental and computational approaches. The results demonstrated that the concrete with marine shells achieved a compressive strength of 32.4 MPa, 26.5% higher than conventional concrete, and a 1% reduction in weight. In contrast, the recycled rubber concrete exhibited a compressive strength of 22.5 MPa, with a 2 MPa decrease compared to conventional concrete, but a 4.3% reduction in density. Computational analysis revealed that porosity affects Young’s modulus, directly resulting in a reduction in the maximum achievable strength. This work demonstrates that it is feasible to produce eco-friendly structural concrete through the proper integration of industrial waste, contributing to decarbonization and waste valorization.

## 1. Introduction

In the construction sector, sustainable and environmentally friendly measures have been adopted, such as the use of industrial waste or products with extremely slow degradation cycles, contributing to the reduction of global pollution [1,2]. It is important to note that the construction sector accounts for 38% of global CO_2_ emissions, with 16% of these emissions attributable to material extraction, manufacturing, and construction activities [3,4]. Decarbonizing the sector through the development of greener buildings is crucial for advancing toward a low-carbon future [3]. One strategy to integrate waste into construction is through concrete, an essential structural material but with high pollution rates. Concrete, which consumes 17.5 billion tons annually worldwide, uses mineral aggregates that make up 80% of its volume [1]. Incorporating waste as aggregates in concrete could enable the production of eco-friendly concrete, thereby promoting decarbonization in construction, the valorization of environmental waste, and the reduction of non-renewable resource consumption [5]. This integration of waste into concrete is particularly feasible in processes such as modular construction with sustainable cellular concrete blocks [3] and in Construction 4.0 through 3D printing. For these reasons, numerous researchers have explored the incorporation of various types of waste into concrete.

Incorporating waste materials into concrete requires a comprehensive evaluation of the mechanical performance and long-term durability of the new material under various conditions [6], with a focus on the appropriate proportion of mineral aggregate replaced by solid waste. Yang et al. [1] examined the strength of eco-friendly concrete containing nutrient-rich aggregates that promote plant growth, enhancing the vegetative impact of the material. The maximum strength after 28 days of curing was 25.93 MPa, and compressive strength improved as porosity decreased. Zhang et al. [7] investigated how adding biomass particles, such as corn cobs, affects the porosity and strength of eco-friendly concrete for agricultural installations or rural walls. Consistent with the findings of Chen et al. [8], replacing corn cobs with wood increased porosity, reducing compressive strength, but the maximum compressive strength of 7.24 MPa still met the strength requirements for insulation blocks in load-bearing [7]. These phenomena are related to the decline in compressive strength and reduction in the elastic modulus. The reduction in strength is linked to how voids act as stress concentration points, facilitating the nucleation and propagation of cracks under compressive loads in brittle materials. Similarly, these voids reduce the effective area that can bear the applied load, thereby increasing the effective stress on the material and leading to premature failure. Additionally, from a micromechanical perspective, the relationship between porosity and the elastic modulus shows that voids disrupt the continuity of the cement matrix and aggregates, creating regions that do not contribute to the stiffness of the volume, thereby reducing the overall stiffness of the composite [9].

Waste materials are currently being integrated into the life cycle of other engineering products. However, certain types of waste are difficult to valorize or incorporate into product life cycles, resulting in open-ended life cycles. Two problematic and undervalued waste materials are rubber [10] and marine shells [11], both linked to the rapid processes of industrialization, urbanization, economic development, and population growth. These factors have led to the generation of large volumes of solid waste and significant environmental risks. Rubber poses a serious environmental threat due to the expansion of the automotive sector and the increasing demand for rubber products. Global production is projected to reach 2.7 billion tires by 2027, with rubber production expected to rise to 19.1 million tons by 2025, representing a 40% increase from 2010 levels [10,12,13].

Rubber waste is often processed through energy recovery methods like pyrolysis. However, reusing rubber is difficult due to its degradation over time [14,15]. To overcome this, the waste rubber needs to be treated to restore its functionality. Rubber can decrease the density and increase energy absorption when added to materials like concrete, requiring a simple crushing process to replace gravel. Rubber and polypropylene fibers can improve the compressive strength of concrete in appropriate proportions [16]. However, Aziz et al. [17] found a 30% reduction in compressive strength when replacing coarse aggregate with rubber. Other studies confirm that rubber reduces compressive strength but increases deformability [18,19]. The strength reduction is attributed to a weak bond between the material components. Incorporating 15% of rubber by weight into concrete can reduce compressive strength to 33% when replacing coarse aggregate [20]. Increasing rubber content to 40% by weight results in a 50% reduction in strength [18]. The primary cause is the softening of the cement paste due to the rubber [18,20], and the increased cracking due to poor rubber–cement interface and the hydrophobic nature of rubber also contribute to strength reduction. This reduction can be mitigated with additives and by limiting rubber content to 10% [21]. Besides, smaller particles with increased surface area and gas absorption result in greater variation in strength compared to larger particles [18,20].

The volume of marine shell waste continues to increase annually. In China, oyster shell accumulation ranges between 12 and 15 million tons per year [11,22], while mussel shells generate 1.4 million tons of waste [23]. In South Korea, oyster shell waste amounts to 280,000 tons annually [24], and in the United States, shell waste production exceeded 400,000 tons in 2021 [25]. These data highlight the severity of the environmental problem, underscoring the urgent need for alternative waste valorization strategies.

Shell waste can be used in cement production and as a soil additive, but the process of converting it to fine particles or powder is very energy-intensive [26,27]. The conversion of calcium carbonate to calcium oxide requires an oxidative thermal process, which increases energy consumption. Although shells can reduce the need to extract calcium carbonate from mineral sources, thereby reducing the environmental impact of mining, the energy costs must be carefully evaluated. Shells can be used as a coarse aggregate in concrete, requiring only crushing to a uniform size, which significantly reduces energy consumption compared to grinding to powder. Seashells are chemically compatible with cement paste due to their CaCO3 composition, promoting integration and strong interfaces. As a ceramic material, shells may increase the strength of concrete, although this remains unclear.

Using shell sand as a fine aggregate can reduce the compressive strength of concrete by up to 20% [28]. However, it can also increase the porosity of the concrete. Replacing traditional fine aggregate with crushed shells improves flowability but reduces strength [29]. The angular shape of the shells improves distribution within the cementitious matrix and provides mechanical interlock. Shells between 2 mm and 6 mm are effective for permeable concrete pavements, while larger particles (4 mm to 6.3 mm) reduce mechanical strength [30]. In addition, incorporating crushed shells increases air content in concrete, which decreases mechanical properties and increases porosity [31]. Another study found no significant change when using shells as a fine aggregate replacement [32].

Based on a review of the previous literature, only limited research has been found on shells in coarse aggregate concrete. Seashells are an attractive and viable recycling alternative because they are a plentiful waste product that appears chemically compatible with cement paste [33]. This makes them a potentially useful material for creating sustainable construction materials, contributing to waste management and the production of cost-efficient green concrete [34]. Similarly, rubber can provide benefits in terms of deformability and energy absorption when limited to 10% by weight, while efforts should be made to improve the compatibility between rubber and cement paste.

The use of waste materials in durable products, such as concrete, and the potential for recycling or further valorization after disposal are important environmental considerations because they can reduce the carbon footprint and resource consumption associated with construction activities. An investigation into these valorization pathways may prove to be a cost-effective strategy, given that the low demand for these materials tends to result in lower costs compared to other waste materials with well-established or clearly defined valorization pathways.

In this research, the compressive strength of concrete produced using recycled rubber and shells as aggregates for structural applications requiring a minimum compressive strength of 25 MPa was evaluated. Two concrete mixes were developed: one with seashells and one with rubber as a substitute for traditional coarse aggregates. The substitution percentages were set at 10% by weight for rubber and 15% for shells to maintain workability, flowability, and structural capacity. Eucalyptus ash was used as a binder in the rubber-based concrete to improve the rubber-cement bond. The effect of porosity on the modulus of elasticity was analyzed using computational homogenization techniques, allowing the mechanical response to be predicted by simulation. The theoretical density assuming 0% porosity was compared to the experimentally determined porosity to understand the internal structure. The effects of these aggregates on compressive strength and density were evaluated, and the eco-concretes are proposed as viable sustainable construction alternatives.

## 2. Materials and Methods

### 2.1. Materials

To produce eco-friendly concrete, Portland cement meeting ASTM C150 standards [35] was used, along with a superplasticizer additive derived from carboxylic acid. The aggregates included river sand as the fine aggregate and gravel as the coarse aggregate. Using these materials, a reference sample was prepared, considered as traditional concrete, with a specified strength of 25 MPa.

Additionally, recycled rubber, recycled concrete, and marine shells were used as coarse aggregates to produce eco-friendly concrete. Two eco-friendly concrete samples were produced: one composed of recycled concrete and marine shells, and the other of recycled concrete and rubber. The recycled concrete used in the eco-friendly concrete was chosen to produce a low carbon emission material [2]. Samples were manually crushed and then processed in a roller crusher, achieving a coarse aggregate size ranging from 15 to 23 mm. The rubber was sourced from a company that recycles rubber and was provided in particle sizes between 10 and 14 mm. The marine shells were manually crushed, resulting in 9 and 20 mm particles. To achieve this, the samples were passed through sieves to maintain a homogeneous size distribution among the coarse aggregates. To enhance the compatibility between the rubber and the cement paste, all rubber particles were pre-coated with a eucalyptus wood ash paste and allowed to dry at room temperature. This was done to improve the hydrophilic capacity of the surface and increase compatibility with the cement paste. The shells were pre-cleaned to remove any biological residues and minerals that could affect the compressive strength results.

### 2.2. Measure Gradation, Density, and Absorption of Aggregates

To properly formulate concrete, it is essential to understand the characteristics of the materials used. In this regard, a gradation study was conducted using sieves, in accordance with ASTM C136 [36]. This study allowed for the determination of the particle size distribution of the various aggregates and the establishment of the maximum and minimum allowable sizes. The sieves used ranged from No. 100 to 2 inches.

The density and absorption of the aggregates were assessed following the procedures outlined in ASTM C128 [37] for fine aggregates and ASTM C127 [38] for coarse aggregates. These analyses provided the necessary data to determine the amount of water required for the mixtures. Additionally, consistency and workability tests were performed according to ASTM C143 [39] to establish the appropriate water-to-cement ratio. The aggregate quantity was controlled through workability analysis of the concrete, achieving an adequate level of workability for a water/cement ratio of 0.55 with additions of 15 wt.% of marine shells and 10 wt.% of rubber.

### 2.3. Mix Design and Sample Preparation

To achieve the appropriate aggregate proportions for each concrete mix configuration, the gradation and proportion of each constituent were analyzed to ensure they fell within the limits established by ASTM C33 [40]. This approach guarantees the concrete’s quality and durability by ensuring effective cohesion in the mix. Subsequently, the samples were dosed and prepared according to ASTM C192 [41]. In total, twelve samples, each with a diameter of 100 mm and a height of 200 mm, were fabricated for each condition. These samples were cured at a temperature of 22 °C with a relative humidity of 80%, and their compressive strength was evaluated at 7, 28, and 90 days.

### 2.4. Mechanical Testing

For the mechanical characterization, compression tests were conducted following ASTM C39 [42] using a Control AUTOMAX PRO compression testing machine with a resolution of 0.1 kN and a maximum load capacity of 3000 kN. Four samples were tested for each curing period. Before testing, the samples were ground to ensure a better load distribution and reduce the effect of surface defects on the contact faces, thereby minimizing their impact on failure behavior. This was achieved using a sulfur–clay mixture applied at 120 °C.

Nanoindentation tests were performed on the main constituent materials, including river sand (fine aggregate), gravel (coarse aggregate), marine shells, and recycled rubber, to determine their elastic constants. The samples were embedded in epoxy resin and ground using abrasive papers with grits ranging from 240 to 1200, with abundant water used to clean the surface and prevent contamination. Subsequently, the samples were polished with a 0.05 µm alumina suspension for 2 h at a constant rotation speed of 150 rpm.

The nanoindentation tests were conducted using a Hysitron Ti 980 nanoindenter (Bruker Corporation, Billerica, MA 01821, USA), employing trapezoidal loading functions with a loading time of 10 s, a hold time of 20 s, and an unloading time of 10 s. Four indentations per sample were performed with a load of 500 µN. The overall mechanical properties obtained from nanoindentation were obtained from the method proposed by Tuninetti et al. [43] and validated by Rojas-Ulloa et al. [44]. Consequently, the material’s elastic constants were determined using Equation (1):

(1) $$\frac{1-{\upsilon_{s}}^{2}}{E_{s}}=\frac{1}{E_{r}}-\frac{1-{\upsilon_{i}}^{2}}{E_{i}}\;$$

where *υ_s_* and *E_s_* represent the Poisson’s ratio and the modulus of elasticity of the samples, respectively, and *E_r_* is the reduced modulus. *υ_i_* and *E_i_* denote the Poisson’s ratio and the modulus of elasticity of the indenter, respectively. The results obtained for the modulus of elasticity of each material were utilized in the computational homogenization process of the concrete composite material.

### 2.5. Computational Simulation

The composite concrete materials were subjected to finite element homogenization processes using Digimat software to determine the Young’s modulus of the material. This was achieved using a representative elementary volume that considered 0%, 5%, 10%, and 15% porosities. The voxel meshing method was employed, with a voxel size of 80 for each coordinate axis, resulting in a total of 512,000 voxels in the representative elementary volume. Quadratic elements with internal thickening and curvature control were used in the mesh.

Mechanical response simulations were performed using ANSYS Workbench 2019 R1, employing Gauss integration and augmented Lagrange methods for node-to-surface and surface-to-surface contact detection. In addition to the concrete specimen, the simulation included two structural steel support plates to replicate the contact between the specimen and the universal testing machine plates. The contact was simulated as bonded to ensure load transfer. A gradual compressive load was applied to the upper face of the specimen in 15 steps, while all degrees of freedom were constrained on the lower face. The solution was obtained using the iterative incremental Newton–Raphson method. A second-order 3D hexahedral element with 20 nodes was used for the simulation to improve the accuracy of the strain and stress fields, and the element size, determined through mesh convergence studies, was set to 4 mm. This resulted in a model with 231,614 nodes and 55,750 elements. Material damage was simulated using the EKILL code in APDL, allowing the deactivation of elements that exceed strain or stress conditions to represent progressive failure phenomena.

The simulation considered two approaches: the first was an elastic approach based solely on the elastic constants of the material, assuming purely elastic behavior with no plasticity. The second approach involved a nonlinear simulation using the multilinear hardening model.

## 3. Results and Discussion

### 3.1. Physical Characterization and Workability of Sustainable Concrete

**Figure 1:**
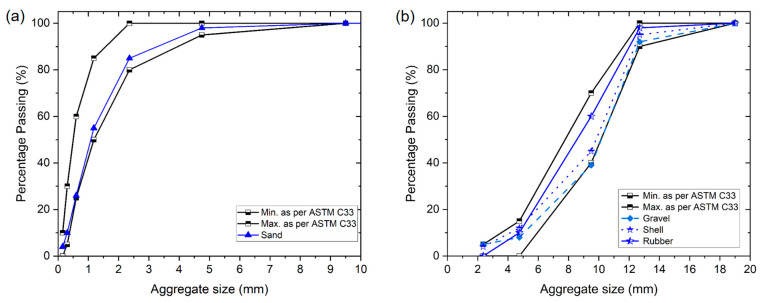
Gradation curves: (**a**) fine aggregate; (**b**) coarse aggregate.

Figure 1 presents the results for various aggregates according to ASTM C33 standards [40], illustrated through gradation curves. Figure 1a shows that the fine aggregate (river sand) is close to the maximum permissible ranges. In contrast, Figure 1b indicates that recycled rubber aligns well with the average values specified by ASTM C33 [40], while marine shells and gravel are near the maximum permissible ranges.

The water absorption results for each aggregate are presented in Table 1. Additionally, workability and consistency tests determined that the optimal water-to-cement ratio is 0.55, allowing for adequate mixing and workability. However, the addition of rubber caused a significant loss in the workability and conformability of the concrete for values above 10 wt.%. In contrast, noticeable changes in workability were observed at a shell content of 15 wt.%. The experimental details of each constituent’s percentage are also shown in Table 1.

**Figure 2:**
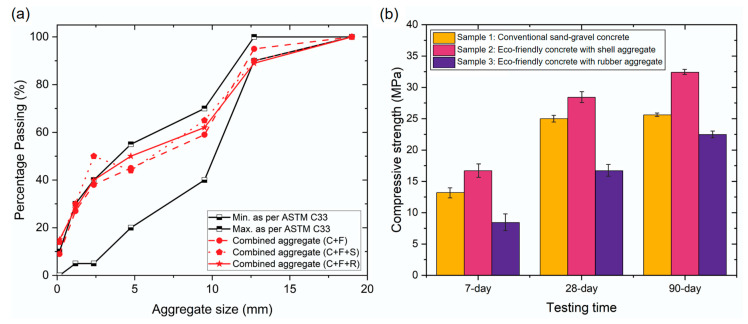
(**a**) Gradation curves for three different mixtures; (**b**) curing time-evolving compressive strength of the three investigated mixtures.

Figure 2a displays the gradation curves for the mixtures corresponding to Sample 1 (traditional concrete), Sample 2 (concrete with marine shells), and Sample 3 (with rubber). The results demonstrate a good fit within the ranges established by ASTM C33 [40], thereby confirming the suitability of the percentages obtained for workability for each sample.

### 3.2. Compressive Strength of Sustainable Structural Concrete Containing Seashells vs. Rubber

Figure 2b shows the mechanical characterization for testing periods of 7, 28, and 90 days during curing. The data reveals a clear trend in the maturation of compressive strength in the concrete, with an expectation of reaching approximately 100% of its maximum strength by 90 days. The compressive strength of Sample 1 stabilizes after 28 days, increasing from 25 MPa to 25.61 MPa at 90 days, representing a 2.44% increase. In contrast, Sample 3 exhibited the lowest compressive strength, reaching 22.46 MPa at 90 days, which indicates a 12.30% reduction in strength compared to Sample 1. However, this translates to a reduction of only 3.15 MPa, which could be offset by its lower density. Finally, Sample 2 demonstrated the highest compressive strength at 90 days, valued at 32.4 MPa. This represents a 26.51% increase in compressive strength compared to traditional concrete. This demonstrates that marine seashells provide greater strength to concrete when used as a replacement for coarse aggregate, indicating that the size or shape of the seashells contributes to higher strength compared to their application as a fine aggregate replacement in concrete.

The primary difference associated with the higher strength achieved by Sample 2, which contains seashells, compared to Sample 3, which contains rubber, is related to the change in stiffness. This change is due to the lower local stiffness contribution provided by the rubber compared to the seashells in the composite material. As a result, the strength achieved by the material is lower for the same level of deformation. Additionally, the crack nucleation capacity in the material at the interface between the aggregate and the matrix is a key factor. Seashells are chemically compatible with the cement paste, primarily due to their compatibility in polarity, generating a stronger interface. Conversely, rubber is a hydrophobic and non-polar material, whereas cement paste is hydrophilic and polar. This incompatibility results in a weaker interface between rubber and cement paste compared to the stronger bond formed between seashells and cement paste. According to Abbas et al. [18], the reduction in compressive strength is attributed to the fact that rubber is a softer material and is not as strong as the traditional aggregates used in concrete. Consequently, the inclusion of rubber reduces the overall strength of the concrete. Additionally, the bonding between the rubber and other concrete ingredients is weak, leading to a higher stress concentration at the rubber aggregates, which further contributes to the decrease in strength properties [18]. Similar trends in compressive strength reduction with rubber have been reported, supporting our findings. Moustafa et al. [45] found that the compressive strength of the concrete mixture with rubber was reduced by up to 15% in comparison to conventional concrete. Thomas et al. [46] determined that the replacement of 10% of natural fine aggregates with crumb rubber resulted in a 29% and 21% reduction in compressive strength at cement ratios of 0.45 and 0.4, respectively. To address the issue of reduced strength, G. Li et al. [47] employed chemical agents to enhance the adhesion of waste rubber to the cement paste. The optimally treated rubber concrete exhibited 4% higher compressive strength than the control concrete. An alternative approach was employed by Onuaguluchi et al. [48], utilizing pre-coated crumb rubber and silica fume in concrete mixtures, which resulted in a 29% enhancement in compressive strength. Incorporating 0.2% basalt fibers and 0.1% polypropylene fibers into rubber concrete resulted in enhanced residual compressive strength, attributed to the reinforcing and crack-arresting effects of the fibers. However, increasing the fiber content led to a comparable decline in rubber concrete’s compressive strength. Replacement of rubber at 10% achieves an acceptable 18% reduction in compressive strength according to Mayha et al. [19]. Lakiar et al. [21] confirmed this by studying aggregate replacement with rubber between 10 and 30%, reaching compression strength reduction between 10 and 52%. 49. Güneyisi et al. [49] proposed a maximum of 15% rubber replacement to obtain about 40% reduction in strength. Similar reductions in strength are also supported by previous research [50,51,52].

The strength loss obtained in the present study is 12.30%, an acceptable variation related to the overall stiffness change in the composite. This change leads to lower strength at the same deformation level as that achieved by stiffer materials. These findings suggest that the rubber coating treatment using eucalyptus ash reduced the hydrophobicity of the rubber, improving its surface affinity with the cement paste and enhancing the interfacial strength of the concrete.

The study findings highlight the feasibility of developing high-strength structural concrete through the utilization of low-value waste materials, including rubber and seashells. This approach integrates the potential of recycled concrete [53], recycled rubber [54], and repurposed seashells [55] as an environmentally responsible solution to mitigate the residual pollution associated with mineral extraction processes [53].

Recycled aggregate concrete supports sustainability by conserving landfill space, managing waste responsibly, reducing aggregate mining, and reducing mining pollution [53]. Xiong et al. [54], using limited quantitative data, demonstrated the environmental advantages of this rubber recycling process for sustainable construction are beyond just reducing greenhouse gas emissions; it also replaces natural aggregates conserving natural resources, mitigates natural disasters, and improves rubber waste disposal [54]. The use of marine shells as a binder has the potential to significantly reduce ordinary Portland cement consumption, thereby lowering CO2 emissions and promoting sustainability through decarbonization [55]. Prior studies and our findings corroborate the feasibility of producing high-performance, sustainable concrete. This approach not only diminishes the density of conventional concrete, facilitating efficient and modular construction, but also repurposes problematic waste materials, such as marine shells and rubber, into a high-quality engineering solution that advances decarbonization in the construction sector.

### 3.3. Failure Behavior of Concrete Compression Samples

**Figure 3:**
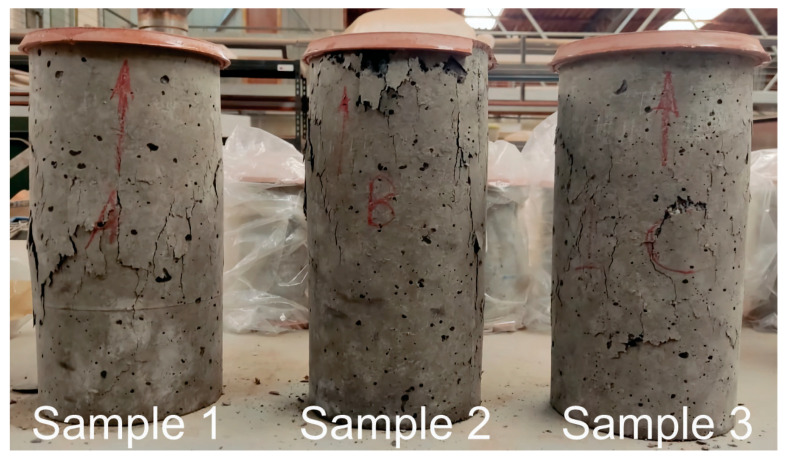
Failure modes of samples at 90 days.

The failure patterns of the tested samples were classified as types 1 and 3 according to ASTM C39 standards (Figure 3). Sample 1 and Sample 2 exhibited fragment detachment, while Sample 3 largely retained its integrity, showing multiple fine cracks. This behavior may be attributed to the reduction in stress concentration at rubber aggregate regions, which acts as a damping effect and results in high energy dissipation capacity. Sample 2 demonstrated a less severe failure compared to Sample 1. This is likely due to the shape and size of the shell aggregate relative to the gravel aggregate, which reduces crack nucleation points and thereby decreases the damage rate. Failure pattern type 1 exhibits a more abrupt loss in load-bearing capacity, making it more susceptible to the formation and propagation of localized cracks. From a durability standpoint, this type of failure can be more critical in environments with cyclic loads, as it accelerates long-term deterioration. In contrast, failure pattern type 3 shows a more uniform stress distribution and a greater deformation capacity, suggesting better performance in environments subjected to cyclic loads.

### 3.4. Overall Elastic Properties of Constituents from Nanoscale Testing

The overall results obtained from nanoindentation on the samples using the method proposed in previous studies [43,44] are detailed in Table 2. It can be observed that the materials with the highest modulus of elasticity are gravel and shells, which are coarse aggregates. Based on these results, it can be deduced that the higher strength achieved by Sample 2 is not due to the greater stiffness of the material but rather to the reduced aggregate size, which decreases the crack initiation points. The results for Sample 3 show a low modulus of elasticity, indicating that, depending on the aggregate size, it acts as a damping element. This explains the reduction in crack thickness and the lower crack nucleation in Sample 3.

### 3.5. Predicting Concrete Behavior from Homogenized Representative Volume Elements

**Figure 4:**
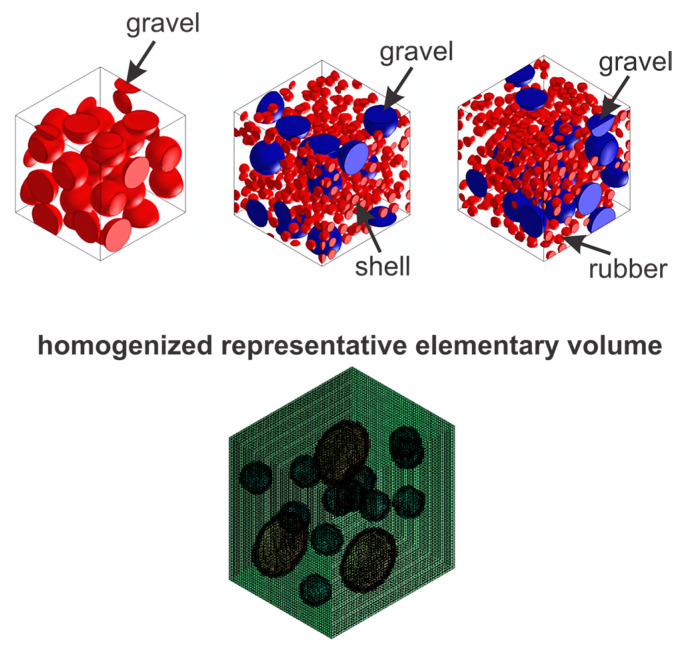
Graphical representation of the homogenization process in the concretes analyzed in this study.

**Figure 5:**
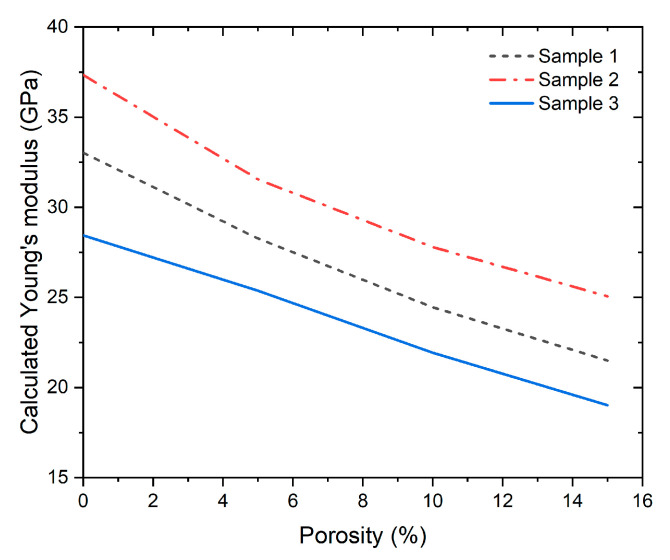
Elasticity modulus calculated through homogenization as a function of porosity.

Figure 4 displays the representative volume elements (RVE) for each sample, along with a schematic representation of the homogenized RVE using an 80-voxel mesh for each structural axis, assuming the absence of porosities. However, it is well known that the aging period of concrete, the water/cement ratio, and compaction directly affect its structural quality and mechanical response due to the presence of internal porosities. The results of the homogenization process considering porosities are detailed in Figure 5.

The results show a clear sensitivity of the elasticity modulus with porosity using the homogenization method (Figure 5). Chung et al. [62] determined the porosity percentage in concrete using Micro-CT, finding that it ranges between 4.28% and 15.46% with variations in additive dosage. The results indicated that strength decreases with increasing porosity percentage and is also sensitive to the shape and size of individual pores. Similarly, Chen et al. [63] characterized porosities in 3D-printed cementitious materials using X-ray computed tomography, demonstrating that the porosity in their samples was not homogeneous, with local porosities approaching 40% in interface zones. Additionally, they observed that outside of the interface zones, the porosity percentage was homogeneous, ranging from 10% to 15%. Camarra et al. [64] found that conventional concrete exhibits a strength range of 29 to 34 MPa, with an elasticity modulus varying between 17 and 43 GPa. Du Plessis et al. [65] indicated that as cement matures, porosity decreases in the center, though the reduction at the edges is less significant. Lian et al. [66] correlated effective porosity with total porosity in concrete, finding that total porosity ranges from 15% to 30% of the sample.

Based on the above, Figure 5 reflects the sensitivity of the loss of rigidity in the concretes analyzed in this study due to the presence of randomly distributed porosity. It is observed that Sample 2 has the highest calculated Young’s modulus, followed by Sample 1, and finally Sample 3. The elasticity modulus ranges for 15% porosity are between 19 and 28 GPa.

### 3.6. Compressive Stress-Strain Relationship

**Figure 6:**
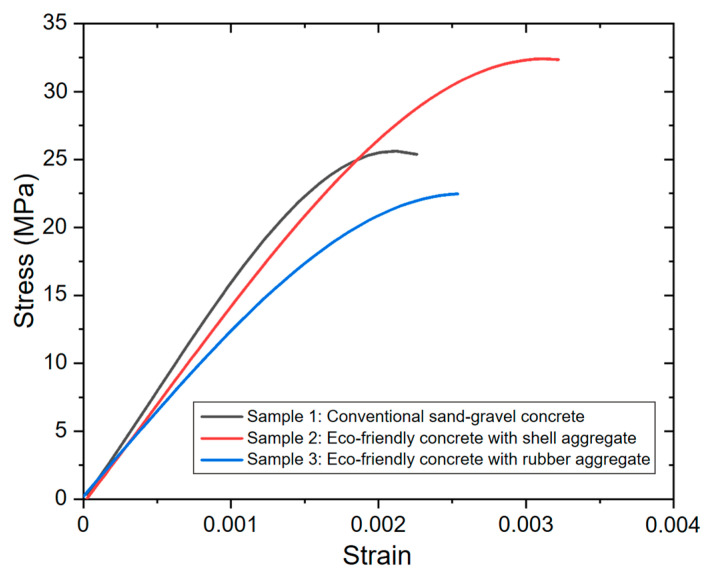
Compressive mechanical response of the studied concretes.

The compression test results are shown in Figure 6, which reveal that Sample 2 exhibits the highest compressive strength compared to Sample 1. These findings highlight the potential for strategically utilizing non-valorized waste in the construction industry to enhance building performance. This material can be used to produce eco-friendly concrete blocks, thereby supporting modular construction. Additionally, Sample 3 shows only a 2 MPa lower compressive strength than conventional concrete, presenting a viable alternative with adequate strength to replace traditional concrete. This demonstrates that the eucalyptus ash coating enhances the chemical compatibility between the rubber and the cement paste, improving the adhesion zone between these components.

The variations observed in the elasticity modulus from the homogenization simulation can be directly linked to the porosity levels in the different concretes studied. Notably, Sample 2 is more prone to porosity, resulting in a material with high permeability. It can also be inferred that, with proper control of the porosity by adjusting the water-to-cement ratio and the level of compaction, the strength could increase by 5.5 MPa for each 1% reduction in porosity [62].

### 3.7. Finite Element-Based Computational Simulation of Axial Compression of Sustainable Concrete

**Figure 7:**
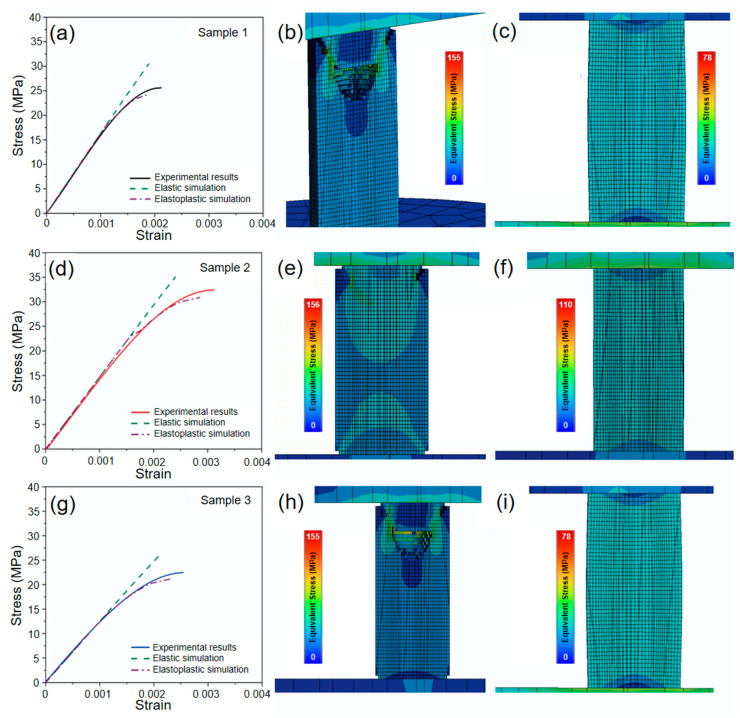
FEM simulation results: (**a**) stress-strain curves for Sample 1 using (**b**) elastic model and (**c**) elastoplasticity; (**d**) stress-strain curves for Sample 2 using (**e**) elasticity and (**f**) elastoplasticity; (**g**) stress-strain curves for Sample 3 using (**h**) elastic simulation results and (**i**) elastoplastic simulation results.

The results of the computational simulation using the Finite Element Method (FEM) are shown in Figure 7. The elastic and plastic simulations fit well within the linear range of the material. Specifically, the plasticity simulation, employing the multilinear hardening model for the three materials studied, demonstrates a suitable trend and fits in the plastic range. However, the achieved strain is lower than that observed experimentally, as damage propagation becomes severe beyond that point, preventing convergence in the simulation (see Figure 7a,d,g). The difference between the deformations reached by the finite element computational model during plasticity and the experimental results is due to the rapid propagation of damage during the simulation, which generates high levels of nonlinearity that prevent the achievement of a numerical convergence state.

Figure 7b shows the results of the elastic simulation for Sample 1, where damage initiates at the top of the specimen, coinciding with the maximum stress point, and propagates inward in a cone-shaped pattern. In contrast, when plasticity is included in the simulation, a slight barreling effect is observed in the compression specimen, which promotes the nucleation of cracks near the center of the specimen, induced by exceeding the material’s allowable displacement. These cracks then propagate towards the points of maximum stress at the upper and lower ends of the specimen.

The elastic simulation results for Sample 2 and Sample 3 (see Figure 7e,h) and the plastic simulation results using the multilinear hardening model (see Figure 7f,i) are consistent with those observed in Sample 1. These findings indicate the presence of two characteristic failure modes in concrete, primarily associated with its stiffness and capacity for plastic deformation. If the material is sufficiently stiff, crack nucleation is expected to occur on the surface and propagate inward (Mode 1). This type of failure has been documented in the work of Howiacki et al. [67], regarding compression tests on standard cylindrical concrete specimens. On the other hand, the type of failure observed in this study corresponds to Mode 2, characterized by crack nucleation due to excessive deformation, propagating towards the upper and lower ends, where maximum stresses are located. This type of failure has been reported in the studies of Ojeda et al. [68], Ma et al. [69], and Li et al. [70] on cylindrical concrete samples.

### 3.8. Correlating Density, Porosity, and Strength of Sustainable High-Strength Structural Concrete

**Figure 8:**
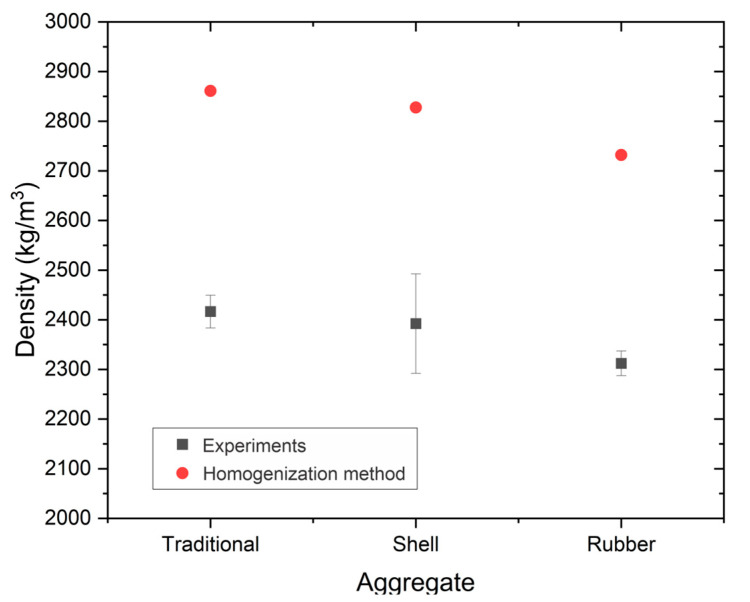
Density results for the concrete samples studied.

Figure 8 displays the experimental density results compared to the theoretical density calculated using the homogenization process. It is noteworthy that the average porosity ranges between 15.35% and 15.53%, indicating that the compaction was uniform across all samples and that the water-to-cement ratio similarly affected the porosity of the samples [71]. However, Sample 2 exhibited the highest deviation, suggesting that its porosity is less stable and can reach up to 18.94% in the most unfavorable case, which impacts the material’s maximum compressive strength.

Regarding the density reduction of Samples 2 and 3 compared to Sample 1, it was found that Sample 3 is 4.31% lighter than Sample 1 under the configurations studied. Additionally, it was confirmed that the compressive strength of Sample 3 is 13.31% lower than that of Sample 1. In contrast, Sample 2 is 1% lighter and 25% stronger than Sample 1. These results support the viability of sustainable construction as an option for reducing solid waste pollution through complex valorization processes. Furthermore, the mechanical performance and advantages such as reduced density enable it to compete effectively with traditional materials.

## 4. Conclusions

This study demonstrates that the incorporation of marine shells into concrete significantly improves its compressive strength, achieving a 26.51% increase compared to conventional concrete. Furthermore, this configuration resulted in a 1% reduction in the weight of the concrete. This suggests that eco-friendly concrete with shell aggregates not only supports sustainable construction practices but also enhances structural performance.

Recycled rubber in concrete caused only a 2 MPa reduction in compressive strength compared to conventional concrete. Despite this decrease in strength, a density reduction of 4.31% was achieved, making it a competitive and environmentally sustainable alternative to conventional concrete, particularly for applications where weight reduction is advantageous, such as in reducing dead loads in buildings.

Computational analysis reveals how porosity affects the modulus of elasticity of eco-friendly concrete, enabling predictions on how changes in the water-to-cement ratio or compaction that reduce porosity will impact the material’s compressive strength.

The results support the integration of complex and difficult-to-recycle industrial wastes such as rubber and marine shells into concrete production as a viable strategy for reducing environmental impact. This approach contributes to the decarbonization of the construction sector and provides a practical solution for managing waste materials while producing high-performance construction materials.

## Figures and Tables

**Table 1:** Water absorption, workability, and consistency results for the sustainable concrete sample preparation.

Materials	Water Absorption (%)	Loose Unit Weight (kg/m^3^)	Sample 1 (C + F) (%)	Sample 2 (C + F + S) (%)	Sample 3 (C + F + R) (%)
Sand (F)	2.3	1695	40	30	35
Gravel (C)	1.9	1691	60	55	55
Shells (S)	1.3	1459	--	15	--
Rubber (R)	0.7	552	--	--	10

**Table 2:** Young’s modulus of different concrete constituents from nanoindentation testing. Poisson’s ratio from the literature.

Materials	Young’s Modulus (GPa)	Poisson’s Ratio
Gravel	75 ± 5	0.23 [56,57]
Shells	60 ± 2	0.32 [58,59]
Cement	33 ± 0.8	0.24 [60]
Rubber	0.15 ± 0.07	0.5 [61]

## Data Availability

The data generated and analyzed during this study are available upon request. Interested parties may contact the corresponding author to access the research data. The availability of data is subject to confidentiality agreements and applicable privacy policies.
